# Unmasking the Silent Threat: Cefepime-Induced Thrombocytopenia

**DOI:** 10.7759/cureus.46518

**Published:** 2023-10-05

**Authors:** Tutul Chowdhury, Sailesh Karki, Parvathy Anitha Rajeev, Annmarie T Sajeev, Binit Aryal, Amulya Bellamkonda, Prava Basnet, Kalpana Panigrahi

**Affiliations:** 1 Internal Medicine, One Brooklyn Health-Interfaith Medical Center, Brooklyn, USA; 2 College of Medicine, American University of Antigua, Coolidge, ATG; 3 Internal Medicine, Hebei Medical University, Shijiazhuag, CHN

**Keywords:** adverse drug reactions, antibacterial activity, broad-spectrum antibiotic, fourth-generation cephalosporin, cefepime

## Abstract

Cefepime, a commonly prescribed fourth-generation cephalosporin, is well-known for its broad-spectrum antibacterial activity. While adverse drug reactions associated with cefepime are well documented, thrombocytopenia as a rare complication has gained attention due to its potential severity. Symptomatic patients present with purpura (bruising), petechiae (small red or purple spots on the skin), and mucosal bleeding. Drug-induced thrombocytopenia can be initiated by myelosuppression by halting platelet formation in the bone marrow or by a drug-induced immune thrombocytopenia reaction. We present a case of a 71-year-old male who experienced thrombocytopenia secondary to cefepime use. We further discussed the underlying mechanisms of cefepime-induced thrombocytopenia, highlighting the need for increased vigilance in monitoring platelet counts during cefepime administration. This case underscores the importance of recognizing and managing this uncommon but potentially life-threatening adverse reaction in clinical practice.

## Introduction

Drug-induced thrombocytopenia (DIT) represents a frequently encountered adverse drug reaction. It can manifest as a substantial hematological challenge, potentially leading to adverse patient outcomes and protracted hospitalization. Among the various drug classes implicated in drug-induced immune thrombocytopenia (DITP), beta-lactam antibiotics, particularly cephalosporins, have been reported as offenders. Cefepime, a fourth-generation cephalosporin is a widely used antibiotic in hospitalized patients [1,2]. Cefepime-induced thrombocytopenia is one such infrequently reported event that is examined in this case report. Here, we present a case of severe thrombocytopenia following the administration of cefepime. We clarify the intricacies of this adverse occurrence, its diagnosis, and its consequences for patient treatment by thoroughly examining a clinical case. 

## Case presentation

We present a 71-year-old male with a past medical history of hypertension, dyslipidemia, traumatic brain injury, post-traumatic seizure disorder, and substance use disorder on methadone. He was brought by emergency medical services (EMS) from home due to lower abdominal pain and low back pain of one-week duration. The abdominal pain was constant, localized to the hypogastrium, aching and cramping type, and non-radiating. While there was no associated chest pain, hematuria, nausea, or vomiting, he reported difficulty in passing urine with an associated increase in frequency, urgency, and dribbling of urine. On examination, blood pressure was 152/80 mmHg with a heart rate of 80 per minute, respiratory rate of 20 per minute, a temperature of 36.4 degrees Celsius, and saturation of 97% on room air. Abdominal examination showed suprapubic tenderness and lower pelvic fullness. There was right costovertebral angle tenderness. Foley’s catheterization was done with the passage of 350 ml of clear urine and a decrease in lower abdominal pain. The initial laboratory test showed blood urea nitrogen (BUN) of 122, creatinine of 14.3, potassium of 8.3, GFR of 3.3, and calcium of 8.4. Labs on admission are listed in Table 1.

**Table 1:** Laboratory test results on Admission WBC: White Blood Cell; Hb: Hemoglobin; MCV: Mean Corpuscular Volume; BUN: Blood Urea Nitrogen; eGFR : Estimated Glomerular Filtration Rate; ALT: Alanine Transaminase; AST :Aspartate Transaminase

Test	Values	Reference Range and Units
WBC	5.3	4.5-11.0 10x3/uL
Hb	8.1	11.0-15.0 g/dL
MCV	86.8	80-100 fL
Platelets	182	130-400 10x3/uL
BUN	122	7.0-18.7 mg/dL
Creatinine	14.3	0.57-1.11 mg/dL
eGFR	3.3	>=90.0
Sodium	135	136-145 mmol/L
Potassium	8.3	3.5-5.1 mmol/L
Bicarbonate	13	22-29 mmol/L
Anion gap	19	mmol/L
Total Bilirubin	0.4	0.2-1.2 mg/dL
ALT	5	10-55 U/L
AST	11	5-34 U/L
Alkaline Phosphatase	427	40-150 U/L
Albumin	3.8	3.5-5.2 g/dL
Phosphorous	5.6	2.3-4.7 mg/dl
Calcium	8.4	8.4-10.2 mg/dL
High sensitivity troponin	58.4	0-17 ng/ml
Prothombin Time	13.6	9.8-13.4 sec
International Normalized ratio	1.14	0.85-1.15
Partial thromboplastin Time	32.1	24.9-35.9 sec
Hemoglobin A1c	4.6	4.8-5.6%
Prostate Specific Antigen	>146	0.00-4.00 ng/ml
Blood culture	Negative	Negative

**Figure 1:**
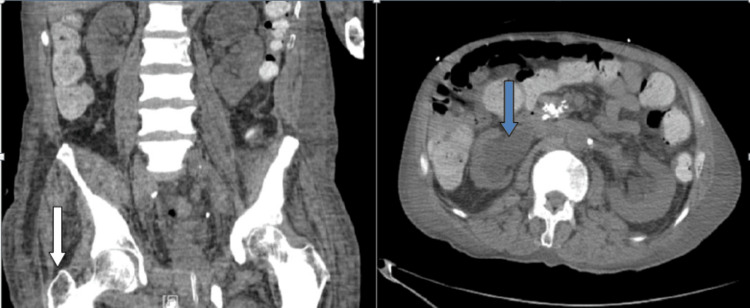
CT abdomen/pelvis showing severe patchy sclerotic changes (white arrow) with kidneys demonstrating hydro-nephrosis bilaterally particularly on the right (blue arrow)

CT abdomen and pelvis showed generalized patchy sclerosis in the bony structures consistent with extensive sclerosing metastatic disease of uncertain etiology (Figure 1). Kidneys demonstrated hydro-nephrosis bilaterally, particularly on the right with a small mid-right ureteral stone. No splenomegaly was noted. 

**Figure 2:**
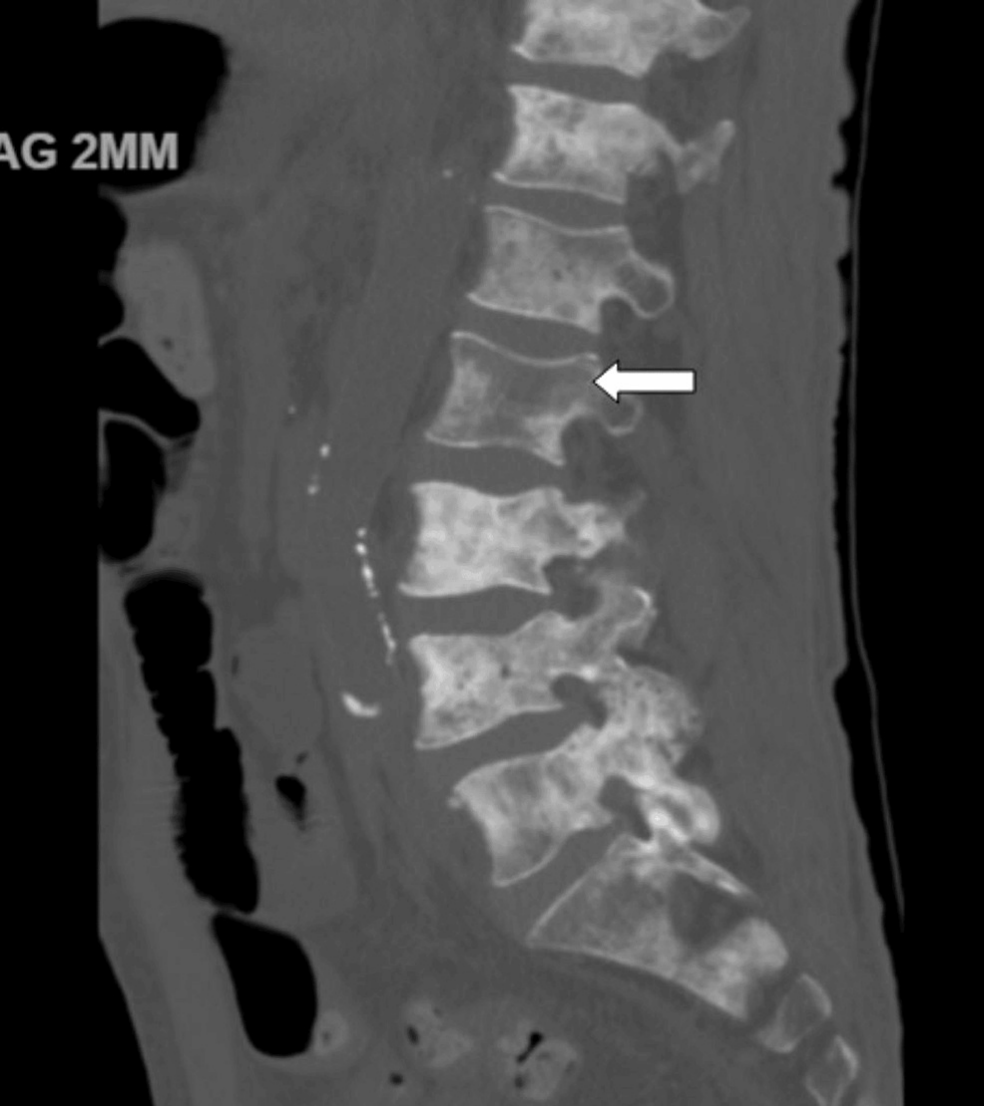
CT lumbar spine showing patchy sclerotic changed (white arrow)

**Figure 3:**
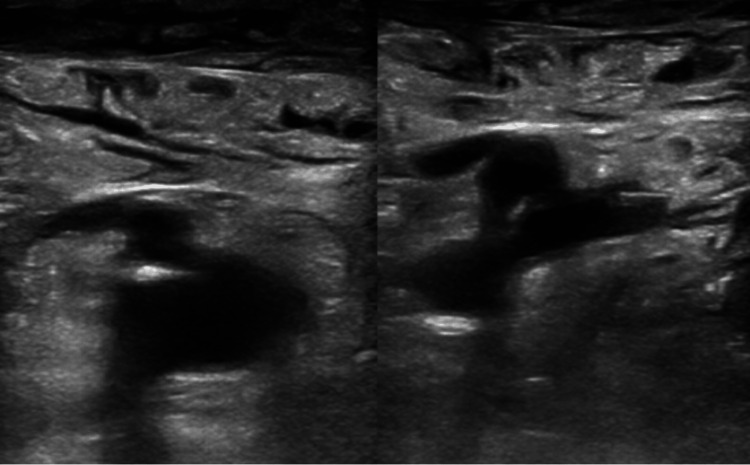
Venous Doppler lower extremities showing no deep vein thrombosis

CT lumbar spine demonstrated diffuse patchy sclerotic metastatic disease (Figure 2). Bilateral lower extremity venous duplex revealed no deep venous thrombosis (Figure 3).

**Figure 4:**
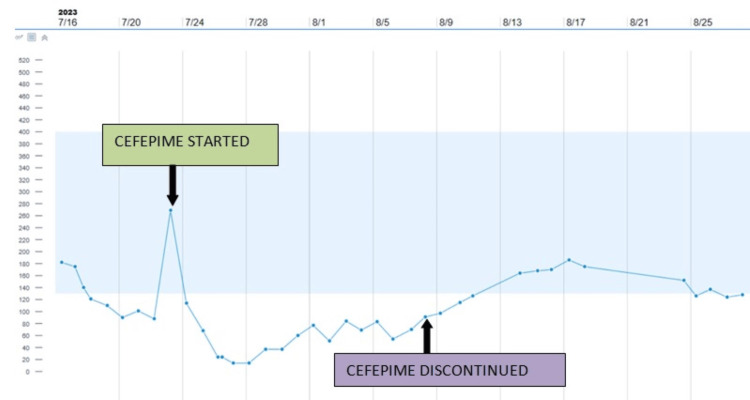
Platelet trending during hospitalization All numbers at left to be multiplied by 10x3 for total platelet count

The patient was diagnosed with acute kidney injury (AKI) likely due to obstructive uropathy, urinary tract infection, and bone metastasis likely from prostate adenocarcinoma. A Foley catheter was placed. The patient was initially started on intravenous ceftriaxone for urinary tract infection (UTI) and switched to cefepime 1 gm intravenous every 24 hours on day 2 of hospitalization. Subsequently, his BUN, creatinine, and potassium improved but his platelets started dropping. Platelet count decreased from 182,000 on admission to 90,000 on day 4 of hospitalization and 24,000 on day 10 of hospitalization. The patient did not have any bleeding despite dropping platelet count. Vitamin B12 was 698 pg/mL (Normal range 180-914 pg/mL). Folate was 4.95 ng/mL (Normal range 5.90-24.80 ng/mL). HIV, hepatitis B, and hepatitis C tests were negative. The 4T score was calculated as 2 points indicating low probability of heparin-induced thrombocytopenia. Cefepime was stopped in view of decreasing platelet count and he was given intravenous immunoglobulin 70 gm daily for two days. Platelet count reached a nadir of 14,000 on day 11 and 12 of hospitalization and started progressively improving from day 12 hospitalization (Figure 4). He was discharged with a platelet count of 152,000 per microliter.

## Discussion

Thrombocytopenia is defined as a reduction in 50% of the platelet count from baseline or less than <100, 000 uL and is common in hospitalized patients. DITP is a prototypical example of a type 2 hypersensitivity reaction. This immunological response is triggered by the presence of a drug, which contains a hapten-drug moiety that binds to a platelet membrane protein. The formation of hapten-protein complexes subsequently initiates an antibody-dependent cascade, resulting in the phagocytosis of megakaryocytes, inflammation, and platelet destruction. [1]

One of the earliest documented instances dates back to 1984 when a patient admitted for cellulitis received intravenous cefamandole, a second-generation cephalosporin, and experienced a notable decline in platelet count along with the development of purpuric skin lesions by the 10th day of treatment [1]. Further investigation through bone marrow aspiration revealed increased cellularity and enhanced megakaryocyte production, indicative of accelerated platelet destruction. Immunofluorescence assays were subsequently performed, confirming the presence of antibodies generated against both second- and third-generation cephalosporins that shared a common thiomethyltetrazole group on the R2 side chain [1]. Remarkably, the patient's condition improved upon administration of cephalexin, a first-generation cephalosporin [1]. This therapeutic success underscores the lack of cross-reactivity among cephalosporin generations, attributable to the high specificity of the antibodies targeting the hapten-protein complexes [2].

The first proven case of cefepime-induced thrombocytopenia was a 35-year-old man with disseminated *Serratia marcescens* infection who subsequently developed severe thrombocytopenia following cefepime administration [1]. A platelet reactive antibody test confirmed cefepime-dependent platelet reactive antibodies, which confirmed the diagnosis. However, the earliest case of cefepime-associated thrombocytopenia was in a 45-year-old male who was admitted to the intensive care unit after allegedly being hit by a large metal bar in the right upper chest and shoulder [3]. It is important to note that the patient suffered from several comorbidities, including AKI and rhabdomyolysis, which may contribute to the subsequent thrombocytopenia after the administration of cefepime [3]. The patient’s platelet count dropped from 102×10^3^/μL to 15×10^3^/μL within a duration of four days. Discontinuation resulted in the patient’s platelet count trending upward to 140x10^3^ after six days [3]. The resulting event on the Naranjo adverse reaction probability scale was a 4, indicating possible causation. Causation was also defined using the criteria in George et al. [3]. It can be inferred that the mechanism was due to the cell-antibody reaction, as the case was compared to the 1984 case, and the duration of thrombocytopenia, drop in platelet count, and resolution of symptoms were similar [3-5]. The only difference was that the patient was asymptomatic in the latter compared to the former (1984) [3].

Despite confirming cephalosporin sensitivity, the incidence of cephalosporin-induced thrombocytopenia remains rare, and these observations have not necessitated a change in the prescribing practices of cephalosporins. Cephalosporin-associated thrombocytopenia has been reported mostly in the second and third generations. However, cefepime, a fourth-generation cephalosporin with broad spectrum coverage, has recently been reported for DITP and should be considered a differential when there is concern for thrombocytopenia. Cefepime, usually administered intravenously, has a reported 70% goal-targeted efficacy for most nosocomial infections [2]. Cefepime is excreted renally, and side effects are seen to occur in patients with renal insufficiency due to the accumulation of the drug. The potentially adverse side effects include neurotoxicity, AKI, and cytopenia. The incidence of cefepime-induced thrombocytopenia is <0.1-1% [2,6].

Primary treatments for cefepime-induced thrombocytopenia, as stated before include discontinuation and treatment with another antibiotic, typically a different cephalosporin. In our patient, cefepime was stopped and no other antibiotic was needed to be given since the course of antibiotics was already completed. Platelet count gradually improved after stopping cefepime. Platelet transfusion is indicated to decrease the severity of bleeding risk in severe thrombocytopenia.

Reports of cefepime-induced thrombocytopenia are rare and inadequately documented. Given the relative novelty of this phenomenon, diminished post-surveillance is common following the administration of fourth-generation cephalosporins for thrombocytopenia. To comprehensively comprehend causation, a profound understanding of cefepime's structure may help establish correlations, if any, between specific drug moieties and antibody formation. Research should explore the duration and quantity of cefepime administration leading to thrombocytopenia and whether the amount of cefepime is influenced by patient comorbidities such as renal dysfunction or critical illness. Cefepime is an antibiotic with a pivotal therapeutic role in bacterial infections. However, the link between cefepime and platelet destruction necessitates further scrutiny to decrease bleeding risk and enhance patient safety. The incidence of cefepime-induced thrombocytopenia underscores the importance of including it as a potential cause when thrombocytopenia is of concern in patient care [2-5].

## Conclusions

While cefepime maintains its pivotal role as an antibiotic for the treatment of bacterial infections, it is essential to exercise caution and consideration of the potential for thrombocytopenia when prescribing it. This report contributes valuable insights to the expanding body of knowledge surrounding this uncommon adverse event, emphasizing its inclusion as a potential etiology of thrombocytopenia within the realm of clinical practice.
